# The effects of green space and physical activity on muscle strength: a national cross-sectional survey with 128,759 Chinese adults

**DOI:** 10.3389/fpubh.2023.973158

**Published:** 2023-05-17

**Authors:** Qiang Feng, Chao-Qun Fan, Jing-Jing Wang, Huan Wang, Dong-Ming Wu, George P. Nassis, Mei Wang, Hai-Jun Wang

**Affiliations:** ^1^Department of Maternal and Child Health, School of Public Health, Peking University, Beijing, China; ^2^Department of National Fitness and Scientific Exercise Research Center, China Institute of Sport Science, Beijing, China; ^3^Physical Education Department–College of Education (CEDU), United Arab Emirates University, Al Ain, United Arab Emirates; ^4^Department of Sports Science and Clinical Biomechanics, SDU Sport and Health Sciences Cluster, University of Southern Denmark, Odense, Denmark

**Keywords:** relative handgrip strength, green space, NDVI, physical activity, additive scale interaction

## Abstract

**Background:**

Muscle strength is closely related to chronic noncommunicable diseases; specifically, a decline in handgrip strength (HS) is predominant globally. Exposure to green space—built environment components used for health intervention—reportedly decreases the risk of certain diseases and all-cause mortality. However, evidence in this area is limited.

**Objective:**

We aimed to explore the association between green space exposure and muscle strength and ascertain the combined effect of physical activity and green space exposure on muscle strength.

**Method:**

Data from 128,759 participants (aged 20–79  years) were obtained using a complex stratified multistage probability cluster sampling design. The green space was assessed as normalized difference vegetation index (NDVI) data for a 500-m buffer zone based on the geolocation information of sampling sites. We used a questionnaire to investigate transportation, occupation, physical activity, leisure-time exercise behaviors, and sedentary time within a usual week of the preceding year. The outcome was low relative HS, defined as HS-to-body weight ratio, and the percentage of men and women with relative HS in the lower third. We defined adequate physical activity as 150  min of moderate-intensity or 75  min of vigorous physical activity per week and calculated the weighted proportion of participants with insufficient physical activity. Categorical variables of NDVI and physical activity were used as exposure variables and their interrelationship was evaluated in a generalized linear mixed model (GLMM) to estimate the odds ratios (ORs) and 95% confidence intervals (95% CI). We measured interaction on an additive or multiplicative scale using a GLMM to test the interaction between green space exposure and physical activity. All analyses were performed for the total sample and subgroups (urban and rural).

**Result:**

The high NDVI group had a lower risk of low relative HS than the low NDVI group (OR [95% CI]: 0.92 [0.88–0.95]). The sufficient physical activity group had a lower risk of low relative HS than the insufficient physical activity group (OR [95% CI]: 0.85 [0.81–0.88]). There was an interactive effect on the additive scale (relative excess risk owing to interaction: 0.29, 95% CI 0.22–0.36, *p <* 0.001) between green space exposure and physical activity.

**Conclusion:**

High NDVI and adequate physical activity were protective factors against low relative HS in Chinese adults. Increasing green space exposure and physical activity together may have a greater potentiating effect on muscle strength improvement than these two protective factors individually. Green spaces should be incorporated into city design or built environments.

## Highlights

– We obtained data from 128,759 consenting Chinese adults.
– We used a complex stratified multistage probability cluster sampling design.
– High NDVI and adequate physical activity protected against low relative HS.
– A combined increase in green space exposure and physical activity may elicit a greater muscle strength improvement than an increase in green space exposure or physical activity alone in people living in rural areas.
– Green spaces may be incorporated in city designs or built environments.

## Introduction

Muscle strength is closely related to chronic non-communicable diseases (1, 2) and all-cause mortality (3); hence, studies on muscle strength are important and have a high public health significance. A decline in handgrip strength (HS) is predominant both globally (4) and in urban and rural Chinese populations (5, 6); thus, more evidence-based solutions are required to address this serious public health problem. Relative HS has recently emerged as a better indicator of overall body muscle strength than absolute muscle strength and might be more comparable among people with different body masses (5, 7, 8).

The World Health Organization (WHO) has created a committee on social determinants of health to improve the essential factors (such as socioeconomic factors, lifestyle, and environmental factors) of communities and individuals, which are key elements of human health. Focusing on the determinants of health and the integration of health into all policies (Health in All Policies) has become a research hotspot in the field of public health (9). Based on the social determinants of the health research framework, lifestyle, and environmental changes (including more green space) may improve muscle strength (10).

With respect to lifestyle, a dose–response relationship has been demonstrated between physical activity and muscle strength, and randomized controlled studies have suggested that physical activity effectively promotes muscle strength in adults (11, 12). In a study with a British cohort of 1,645 participants (778 men and 867 women) born in 1946 physical activity (homemade questionnaire survey) and HS were evaluated, After adjustment for sex, participants with moderate-intensity leisure-time physical activity (MPA) and vigorous leisure-time physical activity (VPA) at the age of 36 years showed higher HS levels at 60–64 years than inactive participants in that study (4). In Korea, insufficient aerobic exercise has been shown to be a risk factor for decreased HS (13). Therefore, increasing the physical activity level could improve muscle strength.

Green spaces are built environment components that can be used for health intervention; they have recently become research hotspots on health outcomes and demonstrated potential health benefits (14, 15). Moreover, green space exposure decreased the risk of cardiovascular disease, diabetes, respiratory disease, obesity, and all-cause mortality. Furthermore, green space exposure might affect muscle strength. McCormack et al. (16) reported that exposure to green space, which is an essential aspect of the Physical Activity Neighborhood Environment Scale, increases self-rated muscle strength. Another study among adolescents showed that the density of parks around schools has a positive effect on the physical fitness score including cardiorespiratory fitness and muscle strength (17). Some studies have demonstrated an association between green space exposure and physical activity; furthermore, at the population level, exposure to green spaces such as parks is crucial for increasing physical activity (18, 19). However, except for these studies, evidence in this area is limited. Further studies are required to clarify whether green space exposure can improve muscle strength; in addition, no study has thoroughly investigated the combined effect of green space exposure and physical activity on muscle strength.

Therefore, we hypothesized that green space exposure can improve muscle strength and physical activity; moreover, green space exposure and physical activity have a combined effect on muscle strength improvement. In this study, we aimed to provide an insight into the association between green space exposure alone, and in combination with physical activity, providing evidence for muscle strength improvement to facilitate health-conscious environmental policy-making.

## Materials and methods

### Study design and participants

Using a complex stratified multistage probability cluster sampling design, we analyzed data from the 2020 Chinese National Survey on Adults’ Fitness, the largest nationally representative survey of civilians in the People’s Republic of China, which was conducted from August to November 2020. The details of recruitment have been described elsewhere (6).

**Figure 1:**
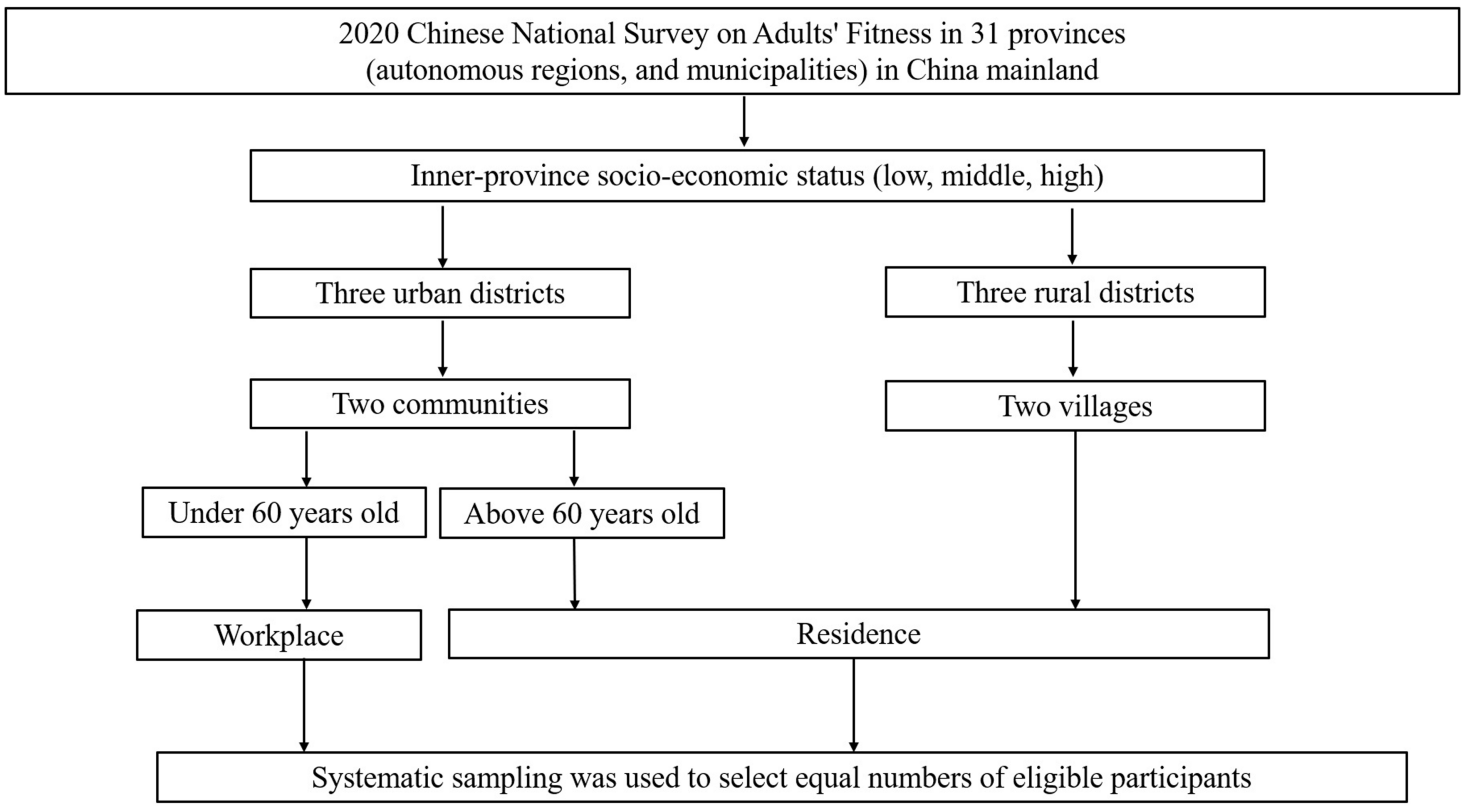
Flow chart of sampling design in the 2020 Chinese National Survey on Adults’ Fitness.

Briefly, 31 provinces, autonomous regions, and municipalities in mainland China were covered in the first stage. In the second stage, three sub-provincial or prefectural-level cities ranked between provinces and counties in the administrative structure of China were randomly selected from each province, based on their economic positions weighted by the gross domestic product assessment, which constitutes the inner-province socioeconomic status (low, middle, and high). Three urban districts (or three rural counties) in each city were selected in the third stage. Three city streets (or rural towns) were chosen for the fourth stage. Two-street community societies (or villages) were selected for the fifth stage. In the final stage, systematic sampling was used to select equal numbers of eligible participants from each workplace or residence to be followed up for at least 3 years. The retirement age in China is 60 years. Participants who were younger than 60 years and living in cities were chosen from the sampling sites based on their workplaces, and participants older than 60 years and living in rural areas were chosen from the sampling sites based on their home addresses. All participants were chosen on the basis of the principles described above and represented Chinese people living in rural and urban areas (Figure 1).

After receiving explanations from trained investigators, participants completed a questionnaire to provide information about demographic characteristics (sex, age, nationality, education level, and career). Each participant signed an informed consent form prior to enrolment. The study protocol was approved by the General Administration of Sports of the People’s Republic of China (20).

### HS and weight measurement

We used a handgrip strength measurement device (Jianmin II, Beijing, People’s Republic of China) to measure the HS in the dominant hand. Before the measurement, the participants held the grip handles with their dominant hand and adjusted the grip width by applying an appropriate grip force. Participants remained upright, with feet naturally separated at shoulder width and arms dropped naturally and inclined at a 10–30° angle from the trunk. After three consecutive tests, we recorded the maximal value for each participant (HS in kg, rounded up to 0.1 kg) with adequate rest intervals (2 min or longer). We used a weight scale (Jianmin II, Beijing, People’s Republic of China) to measure the weight of participants. During the test, the participants wore shorts and bare feet, naturally stood in the center of the scale, kept their body stable, and recorded in kilograms (kg), accurate to one decimal place. The relative HS was calculated using the HS-to-body weight ratio.

### Green space

All site addresses were translated into geolocation information (in terms of longitude and latitude). We excluded all sampling sites with fewer than 10 participants. We used satellite-derived normalized difference vegetation index (NDVI) data for a 500-m buffer zone (NDVI-500), which was determined based on the geolocation information of the sampling sites. In previous studies, exposure to green spaces with smaller buffer zones (250-m buffer zone) had a stronger association with mental health, whereas exposure to green spaces with larger buffer zones (1,000-m buffer zone) had a greater effect on physical activity; hence, we chose a 500-m buffer zone in our analysis (21, 22). We retrieved the NDVI level for each grid cell using Terra Moderate Resolution Imaging Spectroradiometer (MODIS, United States) vegetation indices (MOD13Q1), which were obtained from the United States Geological Survey and generated every 16 days (23). The NDVI value ranges between −1 and + 1, with higher values indicating more green space exposure. As green space exposure influences human behavior in the long term (24, 25), we calculated the mean NDVI for a 12-month period; thus, we set up the start date as 1 year before the survey and the end date as 1 day before the survey. We used the same method described by Cusack et al. (26) and Lin et al. (27) to estimate the NDVI-500 for each sampling site. Moreover, we identified all 16-day composite images that covered the period from the start date to the end date for each sampling site based on the site address. Finally, we summed up the NDVI values and calculated the average as the mean of NDVI-500.

### Physical activity

Through the questionnaire survey, trained investigators collected data on physical activity. We investigated transportation, occupational physical activity, leisure-time exercise behaviors, and sedentary time within a usual week of the preceding year. The questionnaire on physical activity had been described in details in our previous article, which was validated among Chinese adults (*n* = 2,014, age 20–75 years) (6).

We determined different physical activity levels for each participant: low-intensity physical activity, MPA, and VPA. Participants who exercised for more than 10 h/day were excluded. We defined adequate physical activity as achieving 150 or 75 min of MPA or VPA per week, respectively. In addition to physical activity, we collected data on career, education, settlement (urban or rural), and nationality (Han or minority) using a questionnaire.

### Statistical analyses

We used Tukey’s approach (28, 29) to exclude outliers, which were HS and weight values exceeding 1.5 times the interquartile range (method shown in Supplementary Figure S1). We expressed the relative HS as median [interquartile range (IQR)] because the values were not normally distributed. We calculated the low relative HS rate by using the percentage of relative HS. The relative HS of the lower third of male and female participants was taken as the cut-off value.

To explore the relationship between the NDVI and relative HS, we separated the participants into two groups using the median NDVI value in all sample sites: low (rural <0.3327, urban <0.2729) and high (rural ≥0.3327, urban ≥0.2729) NDVI groups.

We used data from the seventh national census released by the China National Bureau of Statistics in 2020, and the survey data were weighted according to sex and 5-year-interval age groups in 31 provinces in mainland China. We then calculated the weighted proportion of participants with sufficient physical activity, which could represent the Chinese population.

The generalized linear mixed model (GLMM) was used to estimate the odds ratios (ORs). The outcome variable was a low relative HS. The categorical variables NDVI and physical activity were considered as separate exposure variables. Additionally, each additional IQR of NDVI and the addition of 150 and 75 min of MPA and VPA per week, respectively, were considered continuous variables.

We presented the results of two statistical models in which the province of each participant was used as a random effect. In the crude model, we only explored the exposure and outcome variables. In the adjusted model, we adjusted for age groups (in 5-year increments from a 20–24 year-old group), sex (male or female), inner-province socioeconomic status (low, middle, or high), nationality (Han or minority), education level (primary school or less, junior high school, senior high school, or university and above), career (technical staff, business people, agricultural personnel, or other occupation), time spent on sedentary activities within 1 week and whether participants had an adequate physical activity or NDVI (low or high) in the year before data collection.

The samples were divided into four groups: high NDVI and sufficient physical activity, high NDVI and insufficient physical activity, low NDVI and sufficient physical activity, and low NDVI and insufficient physical activity. Using the low NDVI and insufficient physical group as the reference group, GLMM was used for OR analysis in the adjusted model. We measured the interaction between green space exposure and physical activity on an additive or a multiplicative scale using the GLMM, wherein NDVI, physical activity, and their product terms were added, and the *p*-value of the product term reflected the interaction under the multiplication scale. Simultaneously, the interaction under the additive scale was determined by calculating the relative excess risk due to interaction (RERI). The delta method facilitates the calculation of the standard error and confidence interval (CI) (30) using Microsoft Excel (Microsoft Corporation, Redmond, WA, United States). All analyses were performed for the total sample population and subgroups (urban and rural sample populations).

In our sampling method, participants aged less than 60 years residing in urban areas were chosen based on their workplaces, and we hypothesized that all participants were not far from home. To test the robustness of the results, we performed two sensitivity analyses. Firstly, we excluded the big cities with populations greater than 5 million people (Beijing, Tianjin, Shanghai, Chongqing, Wuhan, Chengdu, Hangzhou, Nanjing, Zhengzhou, Xian, and Jinan) from the analyses. Secondly, we excluded all samples with weekday transportation time above a cut-off value: walking and riding a bicycle for more than 1 h, riding a motorcycle and driving a car for more than 30 min, and taking a bus for more than 60 min. In the subgroup analysis, we performed stratified analyses between adults aged 20–59 and 60–79 years and between sex groups to test the robustness of our results. In addition, the relative HS was the most studied parameter because it could reflect whole-body strength.

All statistical analyses were performed using R version 3.6.1 (R Foundation for Statistical Computing, Vienna, Austria). Statistical significance was defined as a two-sided *p*-value < 0.05. The results are expressed as the regression coefficient (for continuous variables) or OR (for categorical variables). The weighted rate of adequate physical activity was calculated using the R “survey” package. Data related to green space exposure were downloaded using the R software “modistools” package, and the GLMM used the R software “lm4” and “lmertest” statistical packages. In the additive scale, interactions were determined using the RERI Excel programme developed by Knol et al. (31).

## Results

We invited 158,720 potential participants; of them, 128,759 were enrolled in the study (response rate = 81.1%). Table 1 shows the general characteristics, relative HS values, and physical activity of the study population obtained during the national survey. There was no significant difference in age between the high and low NDVI groups.

**Table 1:** Characteristics of the study population.

	NDVI	
	Low	High
Sample size, n (%)	64,398 (50.0)	64,361 (50.0)
Sex, n (%)		
Male	32,265 (50.1)	32,414 (50.4)
Female	32,133 (49.9)	31,947 (49.6)
Regions, n (%)		
Rural	34,344 (53.3)	34,254 (53.2)
Urban	30,054 (46.7)	30,107 (46.8)
Age group, n (%)		
20–24	5,020 (7.8)	5,139 (8.0)
25–29	5,397 (8.4)	5,341 (8.3)
30–34	5,546 (8.6)	5,407 (8.5)
35–39	5,341 (8.3)	5,258 (8.2)
40–44	5,267 (8.2)	5,473 (8.5)
45–49	5,308 (8.2)	5,471 (8.5)
50–54	4,933 (7.7)	5,253 (8.2)
55–59	4,722 (7.3)	4,974 (7.7)
60–64	5,950 (9.2)	5,476 (8.5)
65–69	5,886 (9.1)	5,590 (8.7)
70–74	5,604 (8.7)	5,654 (8.8)
75–79	5,424 (8.4)	5,262 (8.0)
Inner-province socio-economic status, n (%)		
High	23,521 (37.4)	18,583 (29.0)
Middle	19,901 (31.7)	23,393 (36.5)
Low	19,445 (30.9)	22,091 (34.5)
Education level, n (%)		
Primary school or lower	15,594 (24.6)	16,440 (25.7)
Junior high school	13,574 (21.5)	14,477 (22.6)
Senior high school	11,016 (17.4)	11,390 (17.8)
University and above	23,088 (36.5)	21,703 (33.9)
Career^#^, n (%)		
Technical staff	23,931 (37.9)	22,878 (35.8)
Business people	4,662 (7.4)	3,933 (6.1)
Agricultural personnel	15,789 (25.0)	20,565 (32.1)
Other occupations	18,736 (29.7)	16,600 (25.9)
Nationality, n (%)		
Han	54,803 (85.1)	58,418 (89.2)
Minority	9,595 (14.9)	6,943 (10.8)
Height, Mean (SD)	163.3 (8.6)	161.5 (8.7)
Weight, Mean (SD)	65.4 (11.4)	63.1 (11.2)
Physical activity, min/week, Mean (SD)	162.5 (305.4)	142.5 (282.2)
Sedentary time, min/week, Mean (SD)	637.0 (676.6)	625.6 (688.8)
BMI, kg/m^2^	24.2 (3.5)	24.7 (3.6)
Relative HS^&^, median (IQR)	0.489 (0.183)	0.496 (0.185)

BMI, Body mass index; NDVI, normalized difference vegetation index; SD, standard deviation; IQR, interquartile range. ^#^Age above 60 years (the career was investigated before retirement). ^&^Handgrip strength-to-body weight ratio is presented as median and IQR, whereas other continuous variables are expressed as mean and SD.

Table 2 presents basic information on green space exposure. The median exposure level in the 500-m buffer zone of the whole population was 0.30 (IQR: 0.16). In rural and urban areas, the median exposure levels in the 500-m buffer zones were 0.33 (IQR: 0.18) and 0.27 (IQR: 0.13), respectively. After adjustment for the provincial administrative divisions and economic situation in the province, the NDVI levels in the 500-m buffer zones were significantly higher in rural areas than in cities and towns (*p* < 0.001).

**Table 2:** Basic information related to green space exposure.

	Mean (SD)	IQR	Distribution				
			Min	25th	50th	75th	Max
Total	0.32 (0.11)	0.16	0.08	0.24	0.30	0.39	0.77
Rural	0.35 (0.12)	0.18	0.11	0.26	0.33	0.43	0.77
Urban	0.29^*^ (0.09)	0.13	0.08	0.22	0.27	0.34	0.64

SD, standard deviation; IQR, interquartile range; Min, minimum value; Max, maximum value. ^*^After adjusting for the socio-economic status and 31 provinces, the difference in green space exposure between urban and rural areas is statistically significant, *p* < 0.001.

The basic physical activity levels of the participants are presented in Table 3. The average leisure physical activity times of the total, rural, and urban populations were 152.5 (IQR 240.0), 119.0 (IQR 150.0), and 190.4 (IQR 300.0) min/week, respectively. Moreover, 19,068 (14.8%), 6,865 (10.0%), and 12,203 (20.3%) people reached the recommended levels of physical activity in the total, rural, and urban populations, respectively. The proportion of people who reached the recommended levels of physical activity was significantly higher in the urban than in the rural population (*p* < 0.001).

**Table 3:** Basic information regarding physical activity exposure.

	Mean (SD), (min/week)	IQR, (min/week)	Physical activity group, n (%)	
			Insufficient physical activity	Sufficient physical activity
Total	152.5 (294.2)	240.0	109,691 (85.2)	19,068 (14.8)
Rural	119.0 (264.5)	150.0	61,733 (90.0)	6,865 (10.0)
Urban	190.4 ^*^(320.6)	300.0	47,958 (79.7)	12,203 (20.3^#^)

SD, standard deviation; IQR, interquartile range. *After adjusting for the socio-economic status and 31 provinces, the difference in physical activity level between urban and rural areas is statistically significant, *p* < 0.001 (^#^Chi square test).

Based on the association between the NDVI and relative HS, low NDVI was found to be a risk factor for low relative HS in both models (Table 4). The high NDVI group had a lower risk of low relative HS than the low NDVI group in the total, rural, and urban sample populations in the adjusted model (OR [95% CI]: 0.92 [0.88–0.95], 0.89 [0.85–0.95], and 0.94 [0.89–0.99], respectively). An IQR increase of the NDVI triggered a decrease in the risk of low relative HS in the total and rural sample populations (OR [95% CI]: 0.95 [0.92–0.98] and 0.96 [0.92–0.99], respectively). However, in the urban sample population, an IQR increase of NDVI had no significant effect on the risk of low relative HS (OR [95% CI]: 0.97 [0.93–1.02]).

**Table 4:** Associations between NDVI and relative HS (categorical variable).

	Low NDVI	High NDVI	Increasing one IQR of NDVI	OR (95% CI)	OR (95% CI)
Total (*n* = 128,759)			
Crude	Ref	0.91 (0.88,0.93)	1.07 (1.05,1.10)
Adjusted	Ref	0.92 (0.88,0.95)	0.95 (0.92,0.98)
Rural (*n* = 68,598)			
Crude	Ref	0.87 (0.83, 0.91)	0.93 (0.90, 0.96)
Adjusted	Ref	0.89 (0.85,0.95)	0.96 (0.92, 0.99)
Urban (*n* = 60,161)			
Crude	Ref	1.03 (0.98,1.08)	0.96 (0.92,1.00)
Adjusted	Ref	0.94 (0.89,0.99)	0.97 (0.93,1.02)

CI, confidence interval; NDVI, normalized difference vegetation index; HS, handgrip strength; ref, reference group. Crude Model: used only the provinces as a random effect. Adjusted Model: adjusted for age group, sex, urban and rural areas (only adjusted for urban and rural areas in the total sample), inner-province economic status, nationality, education level, career, sedentary time per week, and sufficient or insufficient physical activity. Provinces were considered the random effect.

Physical activity had a significant relationship with relative HS (Table 5). The sufficient physical activity group had a lower risk of low relative HS than the insufficient physical activity group in the adjusted model (OR [95% CI] of total, rural, and urban sample populations: 0.85 [0.81–0.88], 0.85 [0.80–0.92], and 0.84 [0.79–0.89], respectively). Increasing the MPA level for one IQR of NDVI decreased the risk of low relative HS in the adjusted model (OR [95% CI] of total, rural, and urban sample populations: 0.96 [0.95–0.98], 0.96 [0.94–0.99], and 0.96 [0.94–0.98], respectively). Increasing the VPA level for one IQR of NDVI decreased the risk of low relative HS in the adjusted model (OR [95% CI] of total, rural, and urban sample populations: 0.97 [0.96–0.98], 0.97 [0.96–0.99], and 0.96 [0.95–0.97], respectively).

**Table 5:** Associations between physical activity and relative HS (categorical variable).

	Insufficient physical activity	Sufficient physical activity	Increasing one IQR of MPA	Increasing one IQR of VPA			OR (95% CI)	OR (95% CI)	OR (95% CI)
Total (*n* = 128,759)				
Crude	Ref	0.68 (0.65,0.70)	0.97 (0.96,0.98)	0.89 (0.88,0.90)
Adjusted	Ref	0.85 (0.81,0.88)	0.96 (0.95,0.98)	0.97 (0.96,0.98)
Rural (*n* = 68,598)				
Crude	Ref	0.62 (0.59, 0.66)	0.94 (0.93,0.96)	0.88 (0.87,0.90)
Adjusted	Ref	0.85 (0.80, 0.92)	0.96 (0.94,0.99)	0.97 (0.96,0.99)
Urban (*n* = 60,161)				
Crude	Ref	0.83 (0.79,0.87)	1.01 (1.00,1.03)	0.92 (0.91,0.93)
Adjusted	Ref	0.84 (0.79,0.89)	0.96 (0.94,0.98)	0.96 (0.95,0.97)

CI, confidence interval; HS, handgrip strength; ref, reference group; MPA, moderate-intensity leisure-time physical activity; VPA, vigorous leisure-time physical activity; NDVI, normalized difference vegetation index; OR, odds ratio. Crude Model: used only the provinces as a random effect. Adjusted Model: adjusted for age group, sex, urban and rural areas (only adjusted for urban and rural areas in the total sample), inner-province economic status, nationality, education level, career, sedentary time per week. NDVI and provinces were considered as category variable and random effect, respectively.

Table 6 presents the results of the interaction analysis. In the total sample population, compared with those in the reference group, the ORs [95% CIs] in the low NDVI and sufficient physical activity, high NDVI and insufficient physical activity, and high NDVI and sufficient physical activity groups were 0.85 [0.81–0.91], 0.92 [0.88–0.95], and 0.77 [0.72–0.82], respectively. The joint effect of green space exposure and physical activity was observed on the additive scale (RERI = 0.29, 95% CI 0.22–0.36). However, on a multiplicative scale, the interaction between green space exposure and physical activity was not significant (*p* = 0.713) in the total sample population.

**Table 6:** Interaction between NDVI and physical activity on relative HS.

	Insufficient physical activity		Sufficient physical activity		ORs (95% CI) for HS within strata of NDVI
	N	OR (95% CI)	N	OR (95% CI)	
Total					
Low NDVI	54,644	1.00 (REF)	9,908	0.85 (0.81, 0.91)	0.86 (0.81, 0.91); *p* < 0.001
High NDVI	55,047	0.92 (0.88, 0.95)	9,160	0.77 (0.72, 0.82)	0.85 (0.80, 0.90); *p* < 0.001
ORs (95% CI) for HS within strata of physical activity	0.92 (0.89, 0.96); *p* < 0.001		0.86 (0.79, 0.94); *p* = 0.001		
RERI^#^	0.29 (95% CI: 0.22, 0.36), *p* < 0.001				
*P*_interaction_^*^	*p* = 0.713				
Rural					
Low NDVI	31,014	1.00 (REF)	3,330	0.93 (0.84, 1.02)	0.92 (0.83, 1.01); *p* = 0.089
High NDVI	30,719	0.91 (0.86, 0.96)	3,535	0.71 (0.64, 0.79)	0.80 (0.72, 0.88); *p* < 0.001
ORs (95% CI) for HS within strata of physical activity	0.91 (0.86, 0,97); *p* = 0.002		0.77 (0.64, 0.92); *p* = 0.003		
RERI^#^	0.27 (95% CI: 0.15–0.38), *p* < 0.001				
*P*_interaction_^*^	*p* = 0.016				
Urban					
Low NDVI	23,630	1.00 (REF)	6,578	0.82 (0.76, 0.88)	0.81 (0.75, 0.88); *p* < 0.001
High NDVI	24,328	0.93 (0.88, 0.99)	5,625	0.80 (0.73, 0.87)	0.86 (0.80,0.94); *p* < 0.001
ORs (95% CI) for HS within strata of physical activity	0.94 (0.88, 0.99); *p* = 0.028		0.91 (0.81, 1.03); *p* = 0.132		
RERI^#^	0.32 (95% CI: 0.23, 0.40), *p* < 0.001				
*P*_interaction_^*^	*p* = 0.483				

HS, handgrip strength; NDVI, normalized difference vegetation index; RERI, relative excess risk due to interaction; OR, odds ratio. ^#^Measure of interaction on the additive scale. ^*^Measure of interaction on the multiplicative scale. ORs are adjusted for age group, sex, urban and rural areas (only adjusted for urban and rural areas in the total sample), inner-province economic status, nationality, education level, career, and sedentary time per week. Provinces were considered as the random effect.

In the rural sample population, compared with those in the reference group, the ORs [95% CIs] in the other three groups were 0.93 [0.84–1.02], 0.91 [0.86–0.96], and 0.71 [0.64–0.79]. The joint effect of green space exposure and physical activity was detected on the additive scale (RERI = 0.27, 95% CI 0.15–0.38). On the multiplicative scale, the interaction between green space exposure and physical activity was also significant (*p* = 0.016) in the rural sample population.

In the urban sample population, compared with those in the reference group, the ORs [95% CIs] in the other three groups were 0.82 [0.76–0.88], 0.93 [0.88–0.99], and 0.80 [0.73–0.87]. The combined effect of green space exposure and physical activity was observed on the additive scale (RERI = 0.32, 95% CI 0.23–0.40). However, on the multiplicative scale, the interaction between green space exposure and physical activity was not significant (*p* = 0.483) for the urban sample population.

The estimated joint effect of green space exposure (NDVI) and physical activity was more significant than the product of the estimated effects of green space exposure (NDVI) and physical activity.

In the sensitivity analyses, after excluding participants living in big cities or far from home, the results were comparable with the main results (Supplementary Tables S1–S6). In the stratified analysis, we stratified the different age groups and sexes (Supplementary Tables S7, S8), and the results were in agreement with the main results.

## Discussion

This study found that low NDVI and insufficient physical activity were associated with low relative HS in a national cohort of Chinese adults. Furthermore, the NDVI and physical activity interacted with relative HS. Among people with insufficient physical activity, environmental parameters, such as the NDVI, significantly affected the relative HS. This finding was based on the large number of nationally representative individuals (128,759 adults) in 2020 in the People’s Republic of China.

Low muscle strength is a risk factor for chronic non-communicable diseases (32). Our study provides evidence that high green space exposure is of public health significance. A decreasing trend in absolute and relative HS from 2000 to 2014 has been previously reported (5, 6). In recent years, there has been a decreasing trend in leisure-time physical activity in China (6, 33), which is probably associated with decreased muscle strength. To the best of our knowledge, this is the first study to explore the association between green space exposure and muscle strength in Chinese adults. According to our findings, if the NDVI increases from low to high, which is achievable in urban construction, the risk of low relative HS could decrease by 11 and 6% in rural and urban areas, respectively. This indicates that more green space exposure provides more health benefits, such as increased muscle strength.

Our study found that every increase in IQR (0.158 units) of NDVI in the 500-m buffer zone decreased the risk of low relative HS by 5% in the total sample population. However, compared with those in other studies related to green space, our study showed low NDVI values in the 500-m buffer zone in Chinese adults (only 0.34 units on average); in the relevant reports of foreign developed countries, the average NDVI was mostly greater than 0.5 or 0.6 (24, 34, 35). This result indicates a gap in the green space coverage of the living environment of Chinese adults compared with that of adults in developed countries, which implies significant room for improvement. Therefore, green space exposure is an effective means of health promotion and should play a more central role in urban construction, physical activities, and muscle strength improvement.

The possible mechanism whereby green space exposure improves muscle strength remains unclear. It has been demonstrated that it is not necessary for people to use green spaces to obtain health benefits. For example, one study reported positive health benefits among children and adolescents who did not actually use green spaces for their activities (36), whereas Yang et al. (37) found that the higher the exposure to green spaces around schools, the lower the risk of visual impairment in Chinese children. Another study in China reported that green space exposure reduces all-cause mortality, to a certain extent, in patients with multi-drug resistant tuberculosis who live in areas with short nights (38). Another possible mechanism is that outdoor fitness equipment (OFE) increases the number of green space visitors (39, 40), thereby increasing their muscle strength. In addition to improving the physical activity level (41, 42), OFE in green spaces enhances cardiopulmonary endurance, muscle strength, balance, and flexibility. According to data released by the Chinese government, from 1997 to 2019, there was a seven-fold increase (from 100,000 to 823,500) in the number of OFE built in communities and parks (20, 43). Our study demonstrated that people with high green space exposure may have a higher probability of using OFE.

In China, uneven economic development has caused significant urban–rural differences (44). Some studies have shown that green space exposure has a positive association with different health outcomes, such as obesity (45, 46) and diabetes (47). Furthermore, most studies have focused on green spaces in urban areas of China (48, 49). However, the type of green space that can affect health in rural areas has not been thoroughly studied. Our findings suggest that more attention should be paid to health interventions for people living in rural areas in China owing to the dramatic changes in their lifestyle. Moreover, our results highlight a difference in the effect size of NDVI between urban and rural areas. Green space exposure is much more crucial for people living in rural areas. The high NDVI and sufficient physical activity groups experienced reductions in low relative HS by 29 and 20% in rural and urban populations, respectively, compared with the low NDVI and insufficient physical activity groups. In addition, we observed the interaction effect of green space exposure and physical activity on both additive and multiplicative scales in the rural population. A possible explanation of the difference between urban and rural populations is the distinct lifestyles of Chinese people living in urban and rural areas. More studies should be conducted to clarify the mechanism by which green space exposure improves muscle strength in people living in rural areas.

Our data show that physical activity and green space exposure have a positive interaction, which is in concordance with the results previously obtained in children and adolescents. James et al. (50) found that natural resources, such as parks, simultaneously reduce sedentary time and increase physical activity and fitness. Although the mechanism underlying the positive interaction is unclear, our findings have a high practical significance because we demonstrated that the health benefits at the population level could result from people participating in more physical activity and creating more green spaces. Moreover, among people with insufficient physical activity, building more green spaces seems to be a possible solution for improving muscle strength.

Previous studies have reported a relationship between physical fitness and green space exposure (51, 52), although the evidence is controversial (53, 54). Notably, we found that green space exposure plays an essential role in improving muscle strength, regardless of whether people follow physical activity guidelines. Among people with insufficient physical activity, high green space exposure may be more beneficial for improving the relative HS. However, it is unclear whether green space exposure has a relationship with other types of physical fitness, such as cardiac fitness. Therefore, further studies are necessary.

We found that 150 min of MPA and 75 min of VPA had almost the same effect in decreasing the risk factor of low relative HS; the WHO recommends 150–300 min of MPA or 75–150 min of VPA per week for adults (55). From the results of this study, performing 150 min of MPA and 75 min of VPA has the same effect on improving muscle strength, which adds to the evidence supporting the WHO physical activity guidelines.

The findings of this study have several important practical implications. In recent years, the reduction in global physical activity levels has become a major global public health problem. The proportion of people with insufficient physical activity in major countries ranges between 23.9 and 33.9% (56). In this study, 14.8% of Chinese adults reached the recommended levels of leisure physical activity. Although the physical activities related to occupation and transportation were not considered, the physical activity levels of Chinese adults were still low compared with global physical activity levels. Promoting the improvement of physical activity levels is a systematic project that is affected by individual, environmental, national, economic, urban, and cultural policies as well as other aspects (57). Therefore, it is difficult to effectively increase the physical activity levels of citizens, especially adults. However, this study found that, even in people with insufficient physical activity, green space exposure improves muscle strength, and there is a potential interaction between green space exposure and physical activity. This finding suggests that the promotion of physical activities and role of environmental construction should be considered when developing health promotion policies. The constant improvement in green spaces in the community and narrowing the gap between China and developed countries in terms of available green spaces have important practical significance in muscle strength improvement.

This study had several strengths. Firstly, this is the first study to explore the association between green space exposure and relative HS in a nationally representative sample in China. Secondly, using big population representative data and proper statistical analysis, we found a robust and reliable positive association between green space and muscle strength. To the best of our knowledge, this is the first study to demonstrate the interaction effect of physical activity and green space on muscle strength among people living in rural areas.

However, this study had several limitations. Firstly, the cross-sectional nature of the national survey precludes causal inferences. Longitudinal studies should be conducted to demonstrate the relationship between green space exposure and relative HS. Secondly, we had access only to workplace addresses in urban areas because of the sampling design. However, we performed different sensitivity analyses, and the results indicated that the potential bias might be insignificant. Thirdly, we collected physical activity data using a self-reported questionnaire, which might have led to recall bias. However, a large number of adults (128,759) filled the questionnaires to reduce the error as much as possible. Future studies should perform objective measurements of physical activity to improve the robustness of this analysis. Finally, we used NDVI as the green space exposure index, which could be affected by the weather. However, we used an average value over 12 months to minimize the effects of weather.

In conclusion, low NDVI and physical inactivity are risk factors for low relative HS in Chinese adults. An increase in green space exposure (from low to high NDVI) decreases the risk of low relative HS. A combined increase in green space exposure and physical activity may elicit a greater muscle strength improvement than an increase in green space exposure or physical activity alone in people living in rural areas. Therefore, green spaces should be considered in city design or built environments.

## Data availability statement

The original contributions presented in the study are included in the article/Supplementary material, further inquiries can be directed to the corresponding authors.

## Ethics statement

The studies involving human participants were reviewed and approved by the General Administration of the Sports of the People’s Republic of China (2019021). The patients/participants provided their written informed consent to participate in this study.

## Author contributions

QF contributed to the study analysis plan, data analysis, and manuscript writing. MW conceived and designed the study. H-JW, C-QF, J-JW, HW, and D-MW contributed to the design of the database and electronic case report forms and collated and cleaned the data. GN contributed to the writing up of the manuscript and has provided a critical review of the manuscript. All authors contributed to the article and approved the submitted version.

## Conflict of interest

The authors declare that the research was conducted in the absence of any commercial or financial relationships that could be construed as a potential conflict of interest.

## Publisher’s note

All claims expressed in this article are solely those of the authors and do not necessarily represent those of their affiliated organizations, or those of the publisher, the editors and the reviewers. Any product that may be evaluated in this article, or claim that may be made by its manufacturer, is not guaranteed or endorsed by the publisher.
