# Supplementary material for: The effects of green space and physical activity on muscle strength: a national cross-sectional survey with 128,759 Chinese adults
Source: Front Public Health. 2023 May 17;11:973158. doi: 10.3389/fpubh.2023.973158 (PMC10230031; doi:10.3389/fpubh.2023.973158)
Supplement: Supplementary file 1 [file Data_Sheet_1.pdf]

Table S1 The associations between NDVI and relative HS excluding the big cities in China

	Low NDVI	High NDVI	Increasing one IQR of NDVI
		OR (95% CI)	OR (95% CI)
Total (n=73, 071)			
Crude	ref	0.82 (0.79, 0.85)	1.03 (1.00, 1.06)
Adjusted	ref	0.87 (0.83, 0.92)	0.89 (0.85, 0.92)
Rural (n=38, 615)			
Crude	ref	0.81 (0.76, 0.86)	0.88 (0.84, 0.91)
Adjusted	ref	0.88 (0.81, 0.95)	0.95 (0.90, 1.00)
Urban (n=34, 456)			
Crude	ref	0.96 (0.90, 1.03)	0.92 (0.87, 0.97)
Adjusted	ref	0.91 (0.85, 0.98)	0.89 (0.84, 0.95)

Notes: HS=handgrip strength; ref=reference group. Crude Model: only use the provinces as a random effect. Adjusted Model: adjusted for age group, sex, urban-rural (only adjust region in the total sample), inner-province economic status, nationality, education level, career, sedentary time per week and physical activity adequate or not, provinces as a random effect.

Table S2 The associations between physical activity and relative HS excluding the big cities in China

	Insufficient physical activity	Adequate physical activity	Increasing one IQR of MPA	Increasing one IQR of VPA
		OR (95% CI)	OR (95% CI)	OR (95% CI)
Total (n=73, 071)				
Crude	ref	0.70 (0.66, 0.73)	0.97 (0.95, 0.99)	0.90 (0.89, 0.92)
Adjusted	ref	0.92 (0.86, 0.97)	0.98 (0.96, 1.00)	0.98 (0.97, 1.00)
Rural (n=38, 615)				
Crude	ref	0.68 (0.62, 0.73)	0.95 (0.93, 0.97)	0.91 (0.88, 0.93)
Adjusted	ref	0.91 (0.82, 1.00)	0.97 (0.94, 1.00)	1.00 (0.98, 1.03)
Urban (n=34, 456)				
Crude	ref	0.88 (0.82, 0.94)	1.02 (1.00, 1.04)	0.94 (0.92, 0.96)
Adjusted	ref	0.91 (0.85, 0.98)	0.99 (0.96, 1.01)	0.97 (0.95, 0.99)

Notes: HS=handgrip strength;ref=reference group; MPA=moderate-intensity leisure physical activity; VPA=vigorous leisure physical activity; Crude Model: only use the provinces as a random effect; Adjusted Model: adjusted for age group, sex, urban-rural (only adjust region in total sample), inner-province economic status, nationality, education level, career, sedentary time per week, NDVI as a category variable, provinces as random effect.

Table S3 The associations between NDVI and relative HS excluding the sample far away from home

	Low NDVI	High NDVI	Increasing one IQR of NDVI
		OR (95% CI)	OR (95% CI)
Total (n=107, 986)			
Crude	ref	0.89 (0.86, 0.92)	1.08 (1.06, 1.11)
Adjusted	ref	0.91 (0.87, 0.94)	0.95 (0.92, 0.98)
Rural (n=59, 654)			
Crude	ref	0.87 (0.84, 0.91)	0.93 (0.91, 0.96)
Adjusted	ref	0.89 (0.84,0.95)	0.97 (0.93, 1.01)
Urban (n=48, 332)			
Crude	ref	1.00 (0.95, 1.05)	0.92 (0.88, 0.96)
Adjusted	ref	0.93 (0.88, 0.98)	0.97 (0.91, 1.02)

Notes: HS=handgrip strength; ref=reference group. Crude Model: only use the provinces as a random effect. Adjusted Model: adjusted for age group, sex, urban-rural (only adjust region in the total sample), inner-province economic status, nationality, education level, career, sedentary time per week and physical activity adequate or not, provinces as a random effect.

Table S4 The associations between physical activity and relative HS excluding the sample far away from home

	Insufficient physical activity	Adequate physical activity	Increasing one IQR of MPA	Increasing one IQR of VPA
		OR (95% CI)	OR (95% CI)	OR (95% CI)
Total (n=107, 986)				
Crude	ref	0.67 (0.64, 0.70)	0.97 (0.96,0.98)	0.89 (0.88,0.90)
Adjusted	ref	0.85 (0.81, 0.89)	0.96 (0.94,0.97)	0.97 (0.95, 0.98)
Rural (n=59, 654)				
Crude	ref	0.64 (0.60, 0.68)	0.95 (0.93, 0.97)	0.88 (0.86, 0.90)
Adjusted	ref	0.87 (0.81, 0.94)	0.96 (0.94, 0.99)	0.97 (0.95, 0.99)
Urban (n=48, 332)				
Crude	ref	0.82 (0.78, 0.87)	1.01 (1.00, 1.03)	0.91 (0.90, 0.93)
Adjusted	ref	0.83 (0.78, 0.88)	0.95 (0.93, 0.97)	0.96 (0.95, 0.98)

Notes: HS=handgrip strength; ref=reference group; MPA=moderate-intensity leisure physical activity; VPA=vigorous leisure physical activity; Crude Model: only use the provinces as a random effect. Adjusted Model: adjusted for age group, sex, urban-rural (only adjust region in the total sample), inner-province economic status, nationality, education level, career, sedentary time per week, NDVI as a continuous variable, provinces as a random effect.

Table S5 The interaction between NDVI and physical activity on relative HS excluding the big cities in China

	Insufficient physical activity		Adequate physical activity		ORs (95% CI) for HS within strata of NDVI
	N	OR (95% CI)	N	OR (95% CI)	
Low NDVI	29,264	1.00 (REF)	4,309	0.94 (0.86, 1.02)	0.94 (0.86,1.03); *p*=0.190
High NDVI	33,954	0.88 (0.83, 0.92)	5,544	0.78 (0.72, 0.86)	0.91 (0.84,0.98); *p*=0.016
ORs (95% CI) for HS within strata of physical activity	0.89 (0.84,0.94); *p*<0.001		0.81 (0.71,0.92); *p*=0.001		
RERI#	0.15 (95%CI: 0.05, 0.26), *p*=0.003;				
$P_{interaction}$*	*p*=0.713				

NOTE: RERI=relative excess risk due to interaction; #Measure of interaction on the additive scale; *Measure of interaction on the multiplicative scale; ORs are adjusted for age group, sex, urban-rural (only adjust region in the total sample), inner-province economic status, nationality, education level, career, sedentary time per week, provinces as a random effect.

Table S6 The interaction between NDVI and physical activity on relative HS excluding the sample far away from home

	Insufficient physical activity		Adequate physical activity		ORs (95% CI) for HS within strata of NDVI
	N	OR (95% CI)	N	OR (95% CI)	
Low NDVI	46,022	1.00 (REF)	7,870	0.85 (0.80, 0.91)	0.86 (0.80,0.92); *p*<0.001
High NDVI	46,628	0.91 (0.87, 0.95)	7,466	0.77 (0.72, 0.83)	0.86 (0.80,0.92); *p*<0.001
ORs (95% CI) for HS within strata of physical activity	0.91 (0.87,0.95); *p*<0.001		0.86 (0.78,0.96); *p*=0.005		
RERI#	0.28 (95%CI: 0.21, 0.36), *p*<0.001;				
$P_{interaction}$*	*p*=0.984				

NOTE: RERI=relative excess risk due to interaction; #Measure of interaction on the additive scale; *Measure of interaction on the multiplicative scale; ORs are adjusted for age group, sex, urban-rural (only adjust region in the total sample), inner-province economic status, nationality, education level, career, sedentary time per week, provinces as a random effect.

Table S7 The stratified analyses of the associations between NDVI and relative HS

	Low NDVI	High NDVI	Increasing one IQR of NDVI
		OR (95% CI)	OR (95% CI)
20-59 (n=73,071)			
Crude	ref	0.92 (0.89, 0.96)	0.97 (0.94, 1.00)
Adjusted	ref	0.93 (0.89, 0.97)	0.96 (0.92, 0.99)
60-79 (n=38,615)			
Crude	ref	0.94 (0.90, 0.99)	1.01 (0.98, 1.05)
Adjusted	ref	0.89 (0.83, 0.96)	0.97 (0.92, 1.02)
Male (n=34,456)			
Crude	ref	0.92 (0.89, 0.96)	1.08 (1.05, 1.11)
Adjusted	ref	0.91 (0.87, 0.96)	0.95 (0.91, 0.99)
Female (n=34,456)			
Crude	ref	0.89 (0.85, 0.93)	1.07 (1.03, 1.10)
Adjusted	ref	0.92 (0.88, 0.97)	0.95 (0.91, 0.99)

Notes: HS=handgrip strength; ref=reference group; Crude Model: only use the provinces as a random effect; Adjusted Model: adjusted for age group, sex, urban-rural (only adjust region in the total sample), inner-province economic status, nationality, education level, career, sedentary time per week, physical activity adequate or not, provinces as a random effect.

Table S8 The stratified analyses of the associations between physical activity and relative HS

	Adequate physical activity	Insufficient physical activity	Increasing one IQR of MPA	Increasing one IQR of VPA
		OR (95% CI)	OR (95% CI)	OR (95% CI)
20-59 (n=73,071)				
Crude	ref	0.84 (0.80, 0.88)	0.98 (0.97, 1.00)	0.96 (0.94, 0.97)
Adjusted	ref	0.86 (0.82, 0.91)	0.97 (0.95, 0.99)	0.97 (0.96, 0.98)
60-79 (n=38,615)				
Crude	ref	0.77 (0.73, 0.83)	0.95 (0.93, 0.96)	0.94 (0.91, 0.96)
Adjusted	ref	0.82 (0.75, 0.90)	0.96 (0.94, 0.98)	0.94 (0.91, 0.97)
Male (n=34,456)				
Crude	ref	0.62 (0.59, 0.65)	0.95 (0.94, 0.97)	0.89 (0.88, 0.90)
Adjusted	ref	0.85 (0.80, 0.90)	0.96 (0.94, 0.98)	0.97 (0.95, 0.98)
Female (n=34,456)				
Crude	ref	0.75 (0.71, 0.79)	0.98 (0.96, 1.00)	0.90 (0.88, 0.93)
Model 1	ref	0.81 (0.76, 0.86)	0.95 (0.93, 0.97)	0.96 (0.94, 0.98)

Notes: HS=handgrip strength; ref=reference group; Crude Model: only use the provinces as a random effect; Adjusted Model: adjusted for age group, sex, urban-rural (only adjust region in the total sample), inner-province economic status, nationality, education level, career, sedentary time per week, NDVI as a continuous variable, provinces as a random effect.

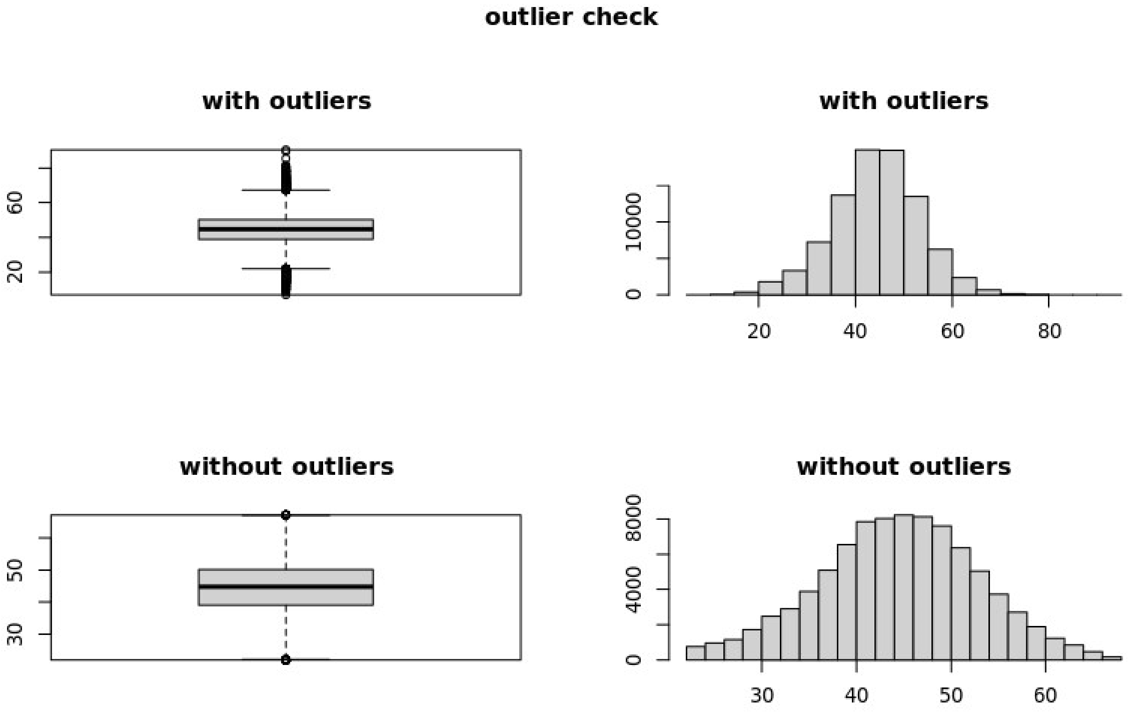
outlier check
with outliers
20
60
with outliers
0
10000
20
40
60
80
without outliers
30
50
without outliers
0
4000
8000
30
40
50
60
